# Dataset of Vietnamese preschool teachers' readiness towards teaching mathematics through experiential education

**DOI:** 10.1016/j.dib.2024.110670

**Published:** 2024-06-26

**Authors:** Manh-Tuan Nguyen, Huyen-Anh Mai, Thi-Tham Tran

**Affiliations:** Faculty of Early Childhood Education, Hanoi National University of Education, 136 Xuan Thuy Road, Cau Giay district, Hanoi 100000, Vietnam

**Keywords:** Early Childhood Education, Inservice Teacher, Mathematics, Experiential Learning, Willingness, Vietnam

## Abstract

Experiential education is an approach that promotes initiative and creativity in students. Many preschool education programs in countries around the world are applying this approach to educational innovation. However, teachers' competencies and readiness to implement experiential education are irreplaceable in determining Mathematics activities' frequency and effectiveness. This dataset surveyed 678 preschool teachers across five cities and provinces in Vietnam from 24 Sep 2023 to 22 Dec 2023. The survey sample was randomly selected, representing different regions of Vietnam. The dataset includes six main sections: (i) Demographics; (ii) Teachers' know-how about Mathematics in early years and experiential education; (iii) Teachers' competencies; (iv) Conditions to implement experiential education; (v) School policies; and (vi) Teachers' readiness to implement experiential education. This dataset can be used by educational policy researchers and early childhood education researchers to study experiential educational practices in early childhood education in Southeast Asian countries or regions of Asia.

Specifications Table

**Table:** 

Subject	Education
Specific subject area	Early Childhood Education, Teacher Development
Type of data	Table
Data collection	An online survey has been distributed throughout randomly selected preschools (from 24 September 2023 to 22 December 2023) in 05 provinces and cities in Vietnam. Researchers emailed the survey to randomly selected principals and asked them to forward it to their teachers voluntarily. At the beginning of the survey, teachers were also informed that their participation was voluntary. Data was gathered using an online survey (Google Form).
Data source location	Five cities and provinces across Vietnam: Hanoi (Latitude 21°1′N, Longitude 105°5′E), Dien Bien (Latitude 21°63′N, Longitude 103°16′E), Bac Giang (Latitude 21°28′N, Longitude 106°20′E), Ninh Binh (Latitude 20°15′N, Longitude 105°58′E), Quang Ninh (Latitude 20°96′N, Longitude 107°04′E).
Data accessibility	Repository name: Mendeley Data Data identification number: nk6kdhd3zm Direct URL to data: https://data.mendeley.com/datasets/nk6kdhd3zm/3 Instructions for accessing these data: Access the website via the link above, this website includes descriptions of the dataset, click on the download button to save the dataset in xlsx format. The dataset citation information is on the right side of this website interface.

## 1 Value of the Data

• This dataset captures preschool teachers' perceptions, capabilities, and readiness to implement experiential education in teaching Mathematics to preschool children.
• This dataset captures Vietnamese preschools' policy and infrastructure toward implementing experiential education in teaching Mathematics to preschool children.
• This dataset provides a comprehensive picture of the readiness to teach various Mathematics content to preschool children through experiential education. The data also provides statistically significant differences in the knowledge and abilities of preschool teachers in different areas.
• This dataset would be helpful for future reference among educational policymakers and practitioners to address the readiness and gaps in implementing experiential education activities for Vietnamese preschools.
• This dataset can be reused by educational policy researchers and early childhood education researchers in studying experiential educational practices in early childhood education in countries or Asia regions.

## 2 Background

Experiential education can be defined as when learners go through four stages that include concrete experiences, reflective observation, abstract conceptualization, and active experimentation to drive learning into practice [1]. Experiential education in the early years engages children in activities, especially in practical contexts where children are encouraged to participate in multi-sensory movements, thereby promoting their problem-solving skills, social-emotional skills, confidence, and empathy [2].

The Vietnamese Early Childhood Education program is a framework built on learning through play and experience. Getting acquainted with Mathematics is an important content in the field of cognitive development for preschool children. Teaching mathematics to preschoolers can be organized through indoor lessons and outdoor activities [3]. However, in the form of indoor lessons, the traditional teaching method begins with the teacher instructing and modeling, then the children practice according to the teacher's instructions, which is a popular method in Vietnam today [4]. Experiential education for preschoolers is still ineffective, activities are almost directed by teachers [5]. Therefore, researching the readiness of preschool teachers to teach Mathematics to children using a new method in Vietnam such as experiential education is necessary to help managers have appropriate policies, contributing to implementing innovation effectiveness in preschool settings.

## 3 Data Description

This dataset focuses on Vietnamese preschool teachers' perceived knowledge and competency to implement experiential education in teaching Mathematics, as well as infrastructure, and policy. The dataset was conducted through an online survey using Google Forms, from 24 September 2023 to 22 December 2023. Participants from randomly selected preschools in five cities and provinces in Vietnam participated voluntarily in this project. After receiving all responses and clarifying the data with 678 validated observations, we exported them as an Excel file (including both *xlsx and *csv formats) for import into SPSS 20. The Excel file (*xlsx) includes two sheets: (i) the complete questionnaires with detailed parameters and (ii) the raw dataset. The questionnaires included seven demographic questions and 53 measurement items. Table 1 contains six fields on the demographics of 678 respondents, except their years of experience, which can be found in the Excel file (column A, sheet 2).

**Table 1:** Demographics of the respondents (*N* = 678).

Variable	Frequency	Percent
***Qualification***	***678***	***100***
Postgraduates	21	3.1
Bachelor's degree	343	50.6
College degree (3 years of training)	259	38.2
Intermediate degree (2 years of training)	55	8.1
***Region***	***678***	***100***
Urban area	238	35.1
Rural area	233	34.4
Mountainous area	207	30.5
***Type of school***	***678***	***100***
Public preschool	461	68.0
Private preschool	171	25.2
Independent child-care group	18	2.7
Family child-care group	28	4.1
***The numbers of children in class***	678	100
Under 10	22	3.2
Greater than or equal to 10 and less than 20	205	30.2
Greater than or equal to 20 and less than 30	299	44.1
Greater than or equal to 30	152	22.4
***Training and working related to experiential education***	***678***	***100***
I have NEITHER participated in experiential education training courses NOR ORGANIZED Mathematics activities using experiential methods	110	16.2
I have NOT participated in experiential education training courses yet, but I HAVE ORGANIZED Mathematics activities using experiential methods	161	23.7
I have participated in experiential education training courses, but I'm NOT CONFIDENT in organizing Mathematics activities using experiential methods	148	21.8
I have participated in training courses, and I'm CONFIDENT in organizing Mathematics activities using experiential methods	259	38.2
***I was trained by…***	***678***	***100***
Provincial-level Department of Education and training	82	12.1
District-level Department of Education and training	200	29.5
My preschool	257	37.9
Others	42	6.2
Not trained	97	14.3

The main questionnaires with 45 items consist of five sections: (i) Teachers' know-how about Mathematics in early years and experiential education (8 items); (ii) Teachers' competencies to implement experiential education in teaching Mathematics for Preschoolers (17 items); (iii) Conditions to implement experiential education (8 items); (iv) School policies and leadership toward the implementation of the experiential education (6 items); and (v) Teachers' readiness to implement experiential education in teaching Mathematics for Preschoolers (6 items). We used various Likert scales of four, five, and six to eliminate the common method bias.

The first set of questions describes Vietnamese preschool teachers' understanding of experiential education and Mathematics for the early years and their competencies in implementing experiential education in teaching Mathematics (Table 2). The participants were asked to self-evaluate their levels of understanding of various concepts and approaches (from No. 8.1 to 8.8), as well as their ability to design the lesson plan, prepare the environment, organize the activities, and enhance preschoolers' learning processes (from No. 9.1 to 9.17).

**Table 2:** Descriptive statistic of Teachers' experiential education know-how and competencies (*N* = 678).

Variables	Range	Min	Max	Mean	Std. Error	Std. Deviation	Variance
<8> What is your level of UNDERSTANDING about the following issues related to experiential education for preschoolers?							
*8.1. The important of Mathematics in the early years*	*3.0*	*1.0*	*4.0*	*3.357*	*.027*	*.700*	*.490*
*8.2. Contents of Mathematics education for preschoolers*	*3.0*	*1.0*	*4.0*	*3.426*	*.026*	*.687*	*.472*
*8.3. Relation of Mathematics to Real-Life*	*3.0*	*1.0*	*4.0*	*3.086*	*.026*	*.682*	*.465*
*8.4. How children learn Mathematics*	*3.0*	*1.0*	*4.0*	*3.392*	*.028*	*.735*	*.540*
*8.5. The theory and philosophy in experiential educations*	*3.0*	*1.0*	*4.0*	*3.022*	*.029*	*.760*	*.577*
*8.6. The process of organizing experiential educational activities (David Kolb, Dewey,…)*	*3.0*	*1.0*	*4.0*	*2.813*	*.034*	*.874*	*.764*
*8.7. Experiential teaching strategies for preschool children*	*3.0*	*1.0*	*4.0*	*3.056*	*.031*	*.802*	*.644*
*8.8. The perspective of experiential education approach in the Vietnamese Early Childhood Education program*	*3.0*	*1.0*	*4.0*	*3.189*	*.026*	*.679*	*.461*
<9> Please rate YOUR SKILLS following each of the listed steps of applying experiential education in teaching Mathematics to preschoolers below:							
*9.1. Choose age-appropriate topics/content*	*5.0*	*1.0*	*6.0*	*2.920*	*.044*	*1.157*	*1.338*
*9.2. Determine requirements that need to be met in accordance with the topic and age*	*5.0*	*1.0*	*6.0*	*2.935*	*.046*	*1.189*	*1.414*
*9.3. Anticipate children's experiential activities and experience time*	*5.0*	*1.0*	*6.0*	*2.925*	*.045*	*1.180*	*1.393*
*9.4. Prepare and organize materials, especially loose parts materials*	*5.0*	*1.0*	*6.0*	*3.055*	*.045*	*1.181*	*1.396*
*9.5. Design of math tools and toys*	*5.0*	*1.0*	*6.0*	*2.987*	*.045*	*1.165*	*1.357*
*9.6. Guide children to explore*	*5.0*	*1.0*	*6.0*	*3.133*	*.046*	*1.203*	*1.448*
*9.7. Instructions for ensuring safety for children*	*5.0*	*1.0*	*6.0*	*3.227*	*.047*	*1.230*	*1.514*
*9.8. Predict the results of children's discoveries*	*5.0*	*1.0*	*6.0*	*3.217*	*.047*	*1.229*	*1.511*
*9.9. Encourage children to pay attention to the content they need to explore*	*5.0*	*1.0*	*6.0*	*3.282*	*.048*	*1.240*	*1.538*
*9.10. Guide children to record the results of their discoveries and experiences*	*5.0*	*1.0*	*6.0*	*3.298*	*.047*	*1.233*	*1.521*
*9.11. Create activities to promote children's learning through inquiry, investigation, thinking, problem-solving, cooperation, communication and creativity*	*5.0*	*1.0*	*6.0*	*3.181*	*.046*	*1.202*	*1.443*
*9.12. Encourage children to share their discoveries*	*5.0*	*1.0*	*6.0*	*3.117*	*.046*	*1.197*	*1.432*
*9.13. Skills to observe children during experiences in learning*	*5.0*	*1.0*	*6.0*	*3.177*	*.046*	*1.196*	*1.431*
*9.14. Ask closed and open questions during the experience*	*5.0*	*1.0*	*6.0*	*3.260*	*.047*	*1.218*	*1.483*
*9.15. Synthesize and generalize knowledge and skills with children*	*5.0*	*1.0*	*6.0*	*3.304*	*.047*	*1.219*	*1.485*
*9.16. Evaluate the process and evaluate the results of children's activities, creating opportunities for children to self-evaluate*	*5.0*	*1.0*	*6.0*	*3.216*	*.046*	*1.192*	*1.422*
*9.17. Guide children to apply knowledge in new situations*	*5.0*	*1.0*	*6.0*	*3.200*	*.046*	*1.197*	*1.434*

Table 3 reports Vietnamese preschools' readiness (from No. 10.1 to 10.8) and organizational supportiveness (from No. 11.1 to 11.6) to implement experiential activities in teaching Mathematics. Those elements are essential to foster teachers' readiness to participate in the experiential activities designing and planning process and the effectiveness of those activities [6].

**Table 3:** Descriptive statistic of infrastructure, policy, and management-related factors toward implementing experiential education in teaching Mathematics (*N* = 678).

Variables	Range	Min	Max	Mean	Std. Error	Std. Deviation	Variance
<10> Please assess the level of satisfaction of the conditions for applying experiential education in teaching Mathematics to preschoolers							
*10.1. Enough time for preparing to teach Mathematics in an experiential way*	*4.0*	*1.0*	*5.0*	*3.854*	*.047*	*1.081*	*1.502*
*10.2. Enough quantity of utensils, toys, and materials*	*4.0*	*1.0*	*5.0*	*3.539*	*.047*	*1.225*	*1.335*
*10.3. Enough quality of toys and materials*	*4.0*	*1.0*	*5.0*	*3.805*	*.044*	*1.155*	*1.463*
*10.4. Enough in terms of number of references*	*4.0*	*1.0*	*5.0*	*3.696*	*.046*	*1.209*	*1.467*
*10.5. Enough in terms of reference quality*	*4.0*	*1.0*	*5.0*	*3.751*	*.047*	*1.211*	*1.464*
*10.6. The number of children in the class is appropriate for the organization of the experience*	*4.0*	*1.0*	*5.0*	*3.664*	*.046*	*1.210*	*1.355*
*10.7. Enough space in the classroom for experiential activities*	*4.0*	*1.0*	*5.0*	*3.556*	*.045*	*1.147*	*1.317*
*10.8. Enough space outside the classroom for experiential activities*	*4.0*	*1.0*	*5.0*	*3.586*	*.044*	*1.157*	*1.339*
<11> Please assess the level of support promotes the application of experiential education in teaching Mathematics to preschoolers							
*11.1. Time to research and organize activities*	*4.0*	*1.0*	*5.0*	*3.583*	*.040*	*1.053*	*1.109*
*11.2. Direct professional support from school administrators/experts (supervision, support, evaluation, professional development, etc.)*	*4.0*	*1.0*	*5.0*	*3.454*	*.040*	*1.038*	*1.078*
*11.3. Support participation in professional development activities outside of school (training, professional development, etc.)*	*4.0*	*1.0*	*5.0*	*3.431*	*.041*	*1.074*	*1.152*
*11.4. Support and invest in funding to apply experiential education in teaching Mathematics for preschoolers*	*4.0*	*1.0*	*5.0*	*3.279*	*.042*	*1.099*	*1.209*
*11.5. Motivate teachers to learn and experiment with the application of experiential education in teaching Mathematics to preschoolers*	*4.0*	*1.0*	*5.0*	*3.381*	*.041*	*1.073*	*1.152*
*11.6. The involvement of educational forces (parents, community, local government, etc.)*	*4.0*	*1.0*	*5.0*	*3.392*	*.044*	*1.133*	*1.285*

There are many contents for children to become familiar with Mathematics, which can be divided into six main subjects: Numbers and counting; Calculations (expressed by adding and subtracting objects); Shape and space; Measurement; Patterns; Data (integrated into other contents) [3]. Table 4 presents Vietnamese preschool teachers' evaluation of the readiness to implement experiential education in teaching Mathematics to preschoolers.

**Table 4:** Descriptive statistic of Teacher's readiness to implement experiential education in teaching Mathematics for preschoolers (*N* = 678).

Variables	Range	Min	Max	Mean	Std. Error	Std. Deviation	Variance
<12> Are you ready to teach Mathematics to preschoolers based on experience in which content areas?							
*12.1. Get acquainted with numbers and counting*	*3.0*	*1.0*	*4.0*	*3.270*	*.022*	*.580*	*.336*
*12.2. Get acquainted with caculations*	*3.0*	*1.0*	*4.0*	*3.260*	*.024*	*.618*	*.382*
*12.3. Get acquainted with shape and space*	*3.0*	*1.0*	*4.0*	*3.227*	*.024*	*.626*	*.391*
*12.4. Get acquainted with measurement*	*3.0*	*1.0*	*4.0*	*3.180*	*.024*	*.620*	*.384*
*12.5. Get acquainted with pattern*	*3.0*	*1.0*	*4.0*	*3.077*	*.025*	*.655*	*.428*
*12.6. Get acquainted with data*	*3.0*	*1.0*	*4.0*	*2.959*	*.027*	*.699*	*.489*

The survey results were analyzed using correlation analyses. Table 5 shows the correlation between two constructs: (i) preschool teachers' understanding of experiential education and Mathematics in early years (No. from 8.1 to 8.8) and (ii) their competencies implementing experiential education in teaching Mathematics for preschoolers (No. from 9.1 to 9.17).

**Table 5:** Correlations table for survey questions.

*Variables*	*8.1*	*8.2*	*8.3*	*8.4*	*8.5*	*8.6*	*8.7*	*8.8*
*9.1*	*−0.031*	*.033*	*.057*	*.039*	*−0.010*	*.000*	*−0.011*	*−0.024*
*9.2*	*−0.031*	*.014*	*.052*	*.026*	*.002*	*.021*	*.021*	*.001*
*9.3*	*−0.037*	*.007*	*.074*	*.015*	*−0.021*	*−0.004*	*.018*	*−0.021*
*9.4*	*−0.040*	*.006*	*.057*	*.021*	*−0.008*	*.016*	*.006*	*−0.026*
*9.5*	*−0.030*	*.009*	*.063*	*.034*	*−0.018*	*.005*	*−0.006*	*−0.019*
*9.6*	*−0.048*	*−0.013*	*.051*	*.004*	*−0.010*	*−0.009*	*−0.002*	*−0.023*
*9.7*	*−0.067*	*.006*	.075*	*.019*	*.017*	*.005*	*.013*	*−0.011*
*9.8*	*−0.063*	*.016*	*.068*	*.022*	*−0.018*	*−0.008*	*−0.020*	*−0.046*
*9.9*	*−0.051*	*.024*	.076*	*.033*	*−0.003*	*.013*	*.015*	*−0.025*
*9.10*	*−0.045*	*.050*	.079*	*.058*	*.024*	*.015*	*.019*	*−0.013*
*9.11*	*−0.040*	*.028*	.080*	*.051*	*.005*	*.003*	*.017*	*−0.013*
*9.12*	*−0.059*	*.013*	*.067*	*.020*	*.002*	*.018*	*.024*	*−0.013*
*9.13*	*−0.044*	*.030*	*.074*	*.023*	*.007*	*.011*	*.019*	*−0.005*
*9.14*	*−0.027*	*.035*	.085*	*.043*	*.007*	*.019*	*.018*	*.000*
*9.15*	*−0.049*	*.018*	*.063*	*.012*	*−0.010*	*.013*	*−0.016*	*−0.027*
*9.16*	*−0.055*	*.007*	*.054*	*.001*	*−0.020*	*−0.011*	*−0.019*	*−0.043*
*9.17*	*−0.027*	*.036*	.089*	*.028*	*.002*	*.008*	*.004*	*−0.027*

*Note:* * *p* < .05 (2-tailed); ** *p* < .01 (2-tailed).

Through the dataset, it is possible to show the differences between preschool teachers in different regions (urban, rural, mountainous areas) in terms of Teachers' know-how about Mathematics in early years and experiential education, Teachers' competencies, Conditions to implement experiential education, School policies, and Teachers' readiness to implement experiential education. For example, the Independent Samples Test analyzes the difference in readiness of preschool teachers in applying experiential education in teaching Mathematics in urban areas (U) and mountainous areas (M) (Table 6).

**Table 6:** The difference in the readiness of preschool teachers in Urban (U) and Mountain (M).

		Levene's Test for Equality of Variances		*t*-test for Equality of Means						
		F	Sig.	T	df	Sig. (2-tailed)	Mean Difference	Std. Error Difference	95 % Confidence Interval of the Difference	
									Lower	Upper
12.1	Equal variances assumed	2.823	.094	2.525	443	.012	.13618	.05394	.03017	.24219
	Equal variances not assumed			2.509	421.242	.012	.13618	.05427	.02950	.24286
12.2	Equal variances assumed	3.013	.083	1.653	443	.099	.09836	.05950	−0.01858	.21530
	Equal variances not assumed			1.656	437.149	.098	.09836	.05940	−0.01838	.21511
12.3	Equal variances assumed	.764	.382	.834	443	.405	.04837	.05799	−0.06560	.16234
	Equal variances not assumed			.835	435.565	.404	.04837	.05795	−0.06553	.16227
12.4	Equal variances assumed	2.541	.112	1.750	443	.081	.10108	.05775	−0.01242	.21459
	Equal variances not assumed			1.748	431.891	.081	.10108	.05784	−0.01259	.21476
12.5	Equal variances assumed	7.020	.008	1.824	443	.069	.11450	.06278	−0.00888	.23789
	Equal variances not assumed			1.833	441.048	.067	.11450	.06246	−0.00826	.23726
12.6	Equal variances assumed	.331	.566	1.602	443	.110	.10671	.06660	−0.02419	.23760
	Equal variances not assumed			1.600	432.650	.110	.10671	.06667	−0.02433	.23775

Paired Samples Test analyzes the difference in preschool teachers' readiness in applying experiential education to teach different Mathematics contents (numbers and counting, calculations, shape and space, measurement, patterns, and data). Table 7 describes the comparison results of each pair of contents.

**Table 7:** The difference in the readiness of preschool teachers in Mathematics contents.

		Paired Differences					T	df	Sig. (2-tailed)
		Mean	Std. Deviation	Std. Error Mean	95 % Confidence Interval of the Difference				
					Lower	Upper			
Pair 1	12.1–12.2	.01032	.40112	.01540	−0.01992	.04057	.670	677	.503
Pair 2	12.1–12.3	.04277	.45436	.01745	.00851	.07703	2.451	677	.014
Pair 3	12.1–12.4	.08997	.45720	.01756	.05549	.12445	5.124	677	.000
Pair 4	12.1–12.5	.19322	.58752	.02256	.14891	.23752	8.563	677	.000
Pair 5	12.1–12.6	.31121	.66716	.02562	.26090	.36152	12.146	677	.000
Pair 6	12.2–12.3	.03245	.44371	.01704	−0.00101	.06591	1.904	677	.057
Pair 7	12.2–12.4	.07965	.45099	.01732	.04564	.11365	4.598	677	.000
Pair 8	12.2–12.5	.18289	.58201	.02235	.13900	.22678	8.182	677	.000
Pair 9	12.2–12.6	.30088	.62520	.02401	.25374	.34803	12.531	677	.000
Pair 10	12.3–12.4	.04720	.40763	.01565	.01646	.07794	3.015	677	.003
Pair 11	12.3–12.5	.15044	.56309	.02163	.10798	.19290	6.957	677	.000
Pair 12	12.3–12.6	.26844	.61149	.02348	.22233	.31455	11.431	677	.000
Pair 13	12.4–12.5	.10324	.57876	.02223	.05960	.14689	4.645	677	.000
Pair 14	12.4–12.6	.22124	.56330	.02163	.17876	.26372	10.227	677	.000
Pair 15	12.5–12.6	.11799	.64375	.02472	.06945	.16654	4.773	677	.000

## 4 Experimental Design, Materials and Methods

To design the research approach, we referred to several concepts about readiness. Readiness includes three parts: emotive attitudinal, cognitive, and behavioral readiness [7]. Readiness is considered as being fully prepared for action. Martin, Budhrani, and Wang (2019) considered knowledge, importance, and ability as the main predictors of readiness [8]. Teachers’ readiness to teach can also be understood as a teacher's condition for teaching [9]. Therefore, knowledge about experiential education, Mathematics knowledge, and competencies of preschool teachers are factors that affect the readiness toward teaching Mathematics through experiential learning for preschool children. The competencies of preschool teachers to organize these activities include starting from the preparation stage for the activity (corresponding to skills from 9.1 to 9.5), the ability to organize activities for children goes through 4 steps in David A. Kolb's experiential learning process (corresponding to skills from 9.6 to 9.17) [10,11].

In addition, external conditions including school policies, social, and facilities are also important conditions that affect preschool teachers' readiness to teach Mathematics through experiences. The lack of facilities, especially spaces, and materials that are safe for children affects the organization of Mathematics and Science activities [12]. Thibaut et al. (2018) also found that continuous support from administrators had an impact on teachers' attitudes toward areas of STEM education (including Mathematics) [13]. The questionnaire with indicators related to policy, social, and facilities factors is referenced from the proposals of Alice Y. Kolb and David A. Kolb [10], Thi-Phuong Hoang et al. [11].

This dataset mainly clarified the dependence of the readiness to implement experiential education in teaching Mathematics for preschool children with related aspects: Self-Knowledge; own proficiency level; operating conditions; and the level of support for implementing experiential education in teaching Mathematics for preschoolers. For instance, an overall regression model that illustrates the relationship between those above factors and preschool teachers' readiness (REA) to implement experiential education in teaching Mathematics to preschoolers can be formed as follows:

REA∼β0+β1*KNO+β2*COM+β3*FAC+β4*LEA+u

We examined factors that support and challenge all teachers in formulating questions regarding the readiness to teach Mathematics to preschoolers. The first aspect (KNO) includes the Teachers' understanding of the concept of Mathematics in early years and experiential education, the theory and philosophy in experiential education, and experiential teaching strategies for preschool children. The second aspect (COM) includes preparation (Choosing age-appropriate topics/content; determining requirements that need to be met by the topic and age; etc.), environment design (preparing and organizing materials, especially loose parts materials; designing Mathematics tools and toys; etc.), organize activities (guiding children to explore; ensuring safety for children; predicting the results of children's discoveries; encouraging children to pay attention to the content they need to explore; etc.), assessment (evaluating the process and evaluate the results of children's activities; creating opportunities for children to self-evaluate). The third aspect (FAC) includes time (prep time; time for carrying out experiential education); quantity and quality of toys; reference materials; the number of children in a class; and time for teaching Mathematics. The last aspect (LEA) includes time to study, organize experiential activities, support from forces inside and outside of school, motivation for teachers to implement experiential education, and funding for applying experiential education in teaching and learning, etc.

## Limitations

The study has certain limitations related to the study sample. The convenient approach of selecting provinces and cities to participate in the survey does not provide a representative sample of preschool teachers in Vietnam, teachers participating in the survey are almost from the public school system, which limits the generalization of the results of this study. The next limitation is that this research only focuses on factors affecting the readiness towards teaching Mathematics through experiential education and does not evaluate the quality of experiential activities in teaching Mathematics to preschoolers.

The limitations and results of this study may suggest some future research directions. This research model can be applied to a random, more representative sample of preschool teachers in Vietnam. Furthermore, explaining the factors that affect preschool teachers' readiness to implement Mathematics teaching through experiential education requires qualitative methods. These important suggestions await contributions from further research.

## Ethics statement

Participation in this dataset is fully anonymized and voluntary. Informed consent was obtained from all participants before they participated in this study. The work was conducted with the approval of the Hanoi National University of Education's Institutional Review Board, Decision No. 134/GCN-ĐHSPHN on 22 Apr 2022.

## CRediT authorship contribution statement

**Manh-Tuan Nguyen:** Conceptualization, Supervision, Writing – review & editing. **Huyen-Anh Mai:** Investigation, Methodology, Software, Data curation. **Thi-Tham Tran:** Methodology, Investigation, Data curation.

## Data Availability

Replication data for project "Implementation of experiential education in teaching Mathematics in Vietnamese preschool" (Original data) (Mendeley Data).
